# Recombinant milk fat globule-EGF factor-8 reduces apoptosis via integrin β3/FAK/PI3K/AKT signaling pathway in rats after traumatic brain injury

**DOI:** 10.1038/s41419-018-0939-5

**Published:** 2018-08-28

**Authors:** Yong-Yue Gao, Zi-Huan Zhang, Zong Zhuang, Yue Lu, Ling-Yun Wu, Zhen-nan Ye, Xiang-Sheng Zhang, Chun-Lei Chen, Wei Li, Chun-Hua Hang

**Affiliations:** 1 Department of Neurosurgery, The Affiliated Drum Tower Hospital, School of Medicine, Nanjing University, Zhongshan Road 321, Nanjing, 210008 Jiangsu Province PR China; 2 Department of Neurosurgery, The First Affiliated Hospital of Wannan Medical College, Wuhu, Anhui Province PR China; 3 Department of Neurosurgery, The Second Affiliated Hospital of Guangzhou Medical University, Guangzhou, Guangdong Province PR China; 4 Department of Neurosurgery, The Affiliated Drum Tower Hospital, Nanjing Medical University, Nanjing, Jiangsu Province PR China

## Abstract

Accumulating evidence suggests neuronal apoptosis has the potential to lead to more harmful effects in the pathological processes following traumatic brain injury (TBI). Previous studies have established that milk fat globule-EGF factor-8 (MFG-E8) provides neuroprotection through modulation of inflammation, oxidative stress, and especially apoptosis in cerebral ischemia and neurodegenerative disease. However, the effects of MFG-E8 on neuronal apoptosis in TBI have not yet been investigated. Therefore, we explored the role of MFG-E8 on anti-apoptosis and its potential mechanism following TBI. In the first set of experiments, adult male Sprague–Dawley (SD) rats were randomly divided into Sham and TBI groups that were each further divided into five groups representing different time points (6 h, 24 h, 72 h, and 7 days) (*n* = 9 each). Western blotting, quantitative real-time PCR, and immunofluorescence staining were performed to identify the expression and cellular localization of MFG-E8. In the second set of experiments, four groups were randomly assigned: Sham group, TBI + Vehicle group, and TBI + rhMFG-E8 (1 and 3 µg) (*n* = 15). Recombinant human MFGE8 (rhMFG-E8) was administrated as two concentrations through intracerebroventricular (i.c.v.) injection at 1 h after TBI induction. Brain water content, neurological severity score, western blotting, and immunofluorescence staining were measured at 24 and 72 h following TBI. In the final set of experiments, MFG-E8 siRNA (500 pmol/3 µl), integrin β3 siRNA (500 pmol/3 µl), and PI3K inhibitor LY294002 (5 and 20 µM) were injected i.c.v. and thereafter rats exposed to TBI. Western blotting, immunofluorescence staining, brain water content, neurological severity score, and Fluoro-Jade C (FJC) staining were used to investigate the effect of the integrin-β3/FAK/PI3K/AKT signaling pathway on MFG-E8-mediated anti-apoptosis after TBI. The expression of MFG-E8 was mainly located in microglial cells and increased to peak at 24 h after TBI. Treatment with rhMFG-E8 (3 µg) markedly decreased brain water content, improved neurological deficits, and reduced neuronal apoptosis at 24 and 72 h after TBI. rhMFG-E8 significantly enhanced the expression of integrin-β3/FAK/PI3K/AKT pathway-related components. Administration of integrin-β3 siRNA and LY294002 (5 and 20 µM) abolished the effect of rhMFG-E8 on anti-apoptosis and neuroprotection after TBI. This study demonstrated for the first time that rhMFG-E8 inhibits neuronal apoptosis and offers neuroprotection. This is suggested to occur through the modulation of the integrin-β3/FAK/PI3K/AKT signaling pathway, highlighting rhMFG-E8 as a potentially promising therapeutic strategy for TBI patients.

## Introduction

Traumatic brain injury (TBI) remains a significant health care issue around the world^1^. Although the primary insult at the moment of impact remains the major outcome determining factor, secondary brain injury caused by subsequent pathological processes may persist for a longer period and aggravate TBI damage^2^. Despite recent developments in understanding the pathological processes of TBI, effective therapeutic strategies are yet to emerge. Searching for an effective treatment that improves the outcome of TBI patients is therefore urgently needed.

Milk fat globule-epidermal growth factor [EGF]-factor 8 (MFG-E8), also known as lactadherin in humans, is a secreted multifunctional glycoprotein originally discovered in mouse milk and the mammary epithelium^3^. Further studies identified MFG-E8 as an intrinsic component of the milk fat globule membrane^4^. MFG-E8 has aroused widespread interest over the last few years, being ascribed a mediator role in cell–cell interactions involved in various biological processes and pathophysiological functions, including the innate immune response^5^, angiogenesis^6^, and fertilization^7^. To this end, several cell types can express MFG-E8, including mammary epithelial cells, macrophages, oligodendrocytes, endothelial cells, as well as intestinal and retinal epithelial cells^8^.

In the central nervous system, MFG-E8 is mainly expressed in microglia, while small amounts of MFG-E8 could also be seen in astrocytes and neurons^9,10^. In previous studies, pharmacological upregulation of MFG-E8 expression was shown to have neuroprotective effects in Alzheimer’s disease (AD)^11^. Furthermore, the administration of recombinant human MFG-E8 (rhMFG-E8) could attenuate cerebral injury through suppression of inflammation and apoptosis in cerebral ischemia^5,12^. Both in vitro and in vivo, MFG-E8 promotes the engulfment of apoptosis cells by acting as a conjunctive molecule that combines with phosphatidylserine and α_v_β3-integrin on apoptotic cells and macrophages, respectively^13–15^. Meanwhile, focal adhesion kinase (FAK), as the downstream molecule of the integrin receptor, activates the downstream effector protein kinase B (AKT) to inhibit apoptosis^16,17^. Additionally, rhMFG-E8 exerts beneficial effects in the models of AD and subarachnoid hemorrhage by reducing oxidative stress through modulation of the integrin receptor and heme oxygenase-1 (HO-1)^11,18^.

To date, the effects of MFG-E8 in TBI have not been investigated. It is hypothesized that MFG-E8 may have a favorable influence during the pathological process of TBI. Therefore, the aim of the present study was to investigate the efficacy of MFG-E8 in a TBI mouse model.

## Materials and methods

### Animals preparation

Adult male Sprague–Dawley (SD) rats weighing 250–300 g were purchased from the Experimental Animal Center of Drum Tower Hospital and housed in a 12 h light/dark cycle room. All rats were allowed free access to food and water under conditions of controlled humidity and temperature (24 ± 0.5 °C). The experimental protocols and procedures were approved by the Institutional Animal Care and Use Committee at Drum Tower Hospital and conformed to the National Institutes of Health (NIH) Guide for the Care and Use of Laboratory Animals.

### Rat TBI model

Experimental TBI models used in this study were performed as described in previous studies^19,20^. Briefly, rats were placed in a stereotaxic frame after intraperitoneal anesthesia with sodium pentobarbital (50 mg/kg). After disinfection, a 2.0 cm midline scalp incision was made and the skull was exposed. On the left parietal cortex, a 6-mm craniotomy was performed through the skull; the center of the hole was 2.5 mm lateral to the midline on the mid-coronal plane. The dura was left intact during the operation. The impact was performed by releasing a 40 g weight onto the dura from a height of 25 cm along a steel shaft, which translated to 1000× g/cm. By this time, the scalp wound was thoroughly disinfected and closed with suture. Sham animals were subjected to the same procedures without injury. Then rats were returned to the cages and maintained at a temperature of 24.0 ± 0.5 °C.

### Experimental design

**Fig. 1:**
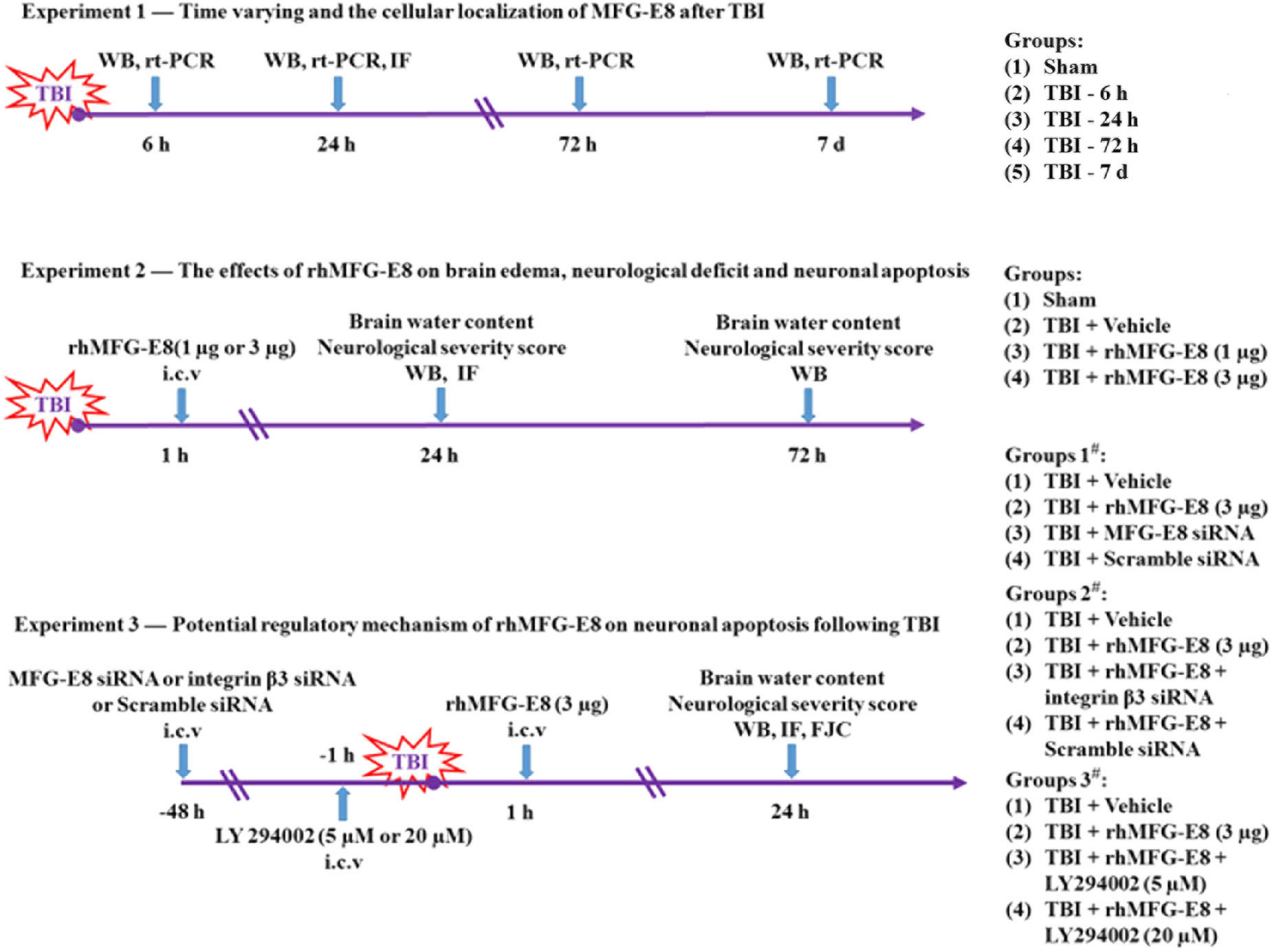
Experimental designs and animal group. TBI traumatic brain injury, WB western blot, rt-PCR quantitative real-time polymerase chain reaction, IF immunofluorescence, h hour, d days, rh-MFG-E8 recombinant human milk fat globule-EGF factor-8, i.c.v. intracerebroventricular, LY294002 PI3K kinase inhibitor

All rats were randomly assigned to the following experiments as described (Fig. 1).

*Experiment design 1*—To examine the expression of MFG-E8 in the cortex of rats after TBI. Rats were randomly assigned to five groups: Sham group and TBI group (6 h, 24 h, 72 h, 7 days) (*n* = 9 each). Six rats of each group were selected randomly for western blot analyses and quantitative real-time polymerase chain reaction and the rest rats for double-immunofluorescence staining.

*Experiment design 2*—To determine the effect of rhMFG-E8 on second brain injury after TBI. Rats were randomly assigned to four groups: Sham group, TBI + Vehicle group, TBI + rhMFG-E8 (1 and 3 µg) (*n* = 15 each). Assessment method including brain water content, neurological severity score, western blot analyses, and double-immunofluorescence staining.

*Experiment design 3*—To explore the potential mechanism of rhMFG-E8 on neuroprotection after TBI. Rats were randomly assigned into the following group: TBI + Vehicle group, TBI + rhMFG-E8, TBI + MFG-E8 siRNA, TBI + Scramble siRNA, TBI + rhMFG-E8 + integrin β3 siRNA, TBI + rhMFG-E8 + Scramble siRNA, TBI + rhMFG-E8 + LY294002 (5 and 20 µM). Assessment method including western blot analyses and double-immunofluorescence staining, brain water content, neurological severity score, and Fluoro-Jade C (FJC) staining.

### Intracerebroventricular administration

Intracerebroventricular (i.c.v.) drug administration was performed as previously described^21,22^. Briefly, rats were placed in a stereotaxic frame after intraperitoneal anesthesia with sodium pentobarbital (50 mg/kg). The needle of a 10-µl Hamilton syringe (Shanghai Gaoge Industry & Trade Co., Ltd., Shanghai, China) was inserted into the right lateral ventricle through a burr hole using the following coordinates: 0.8 mm posterior and 1.5 mm lateral to the bregma, and 3.7 mm below the dural layer. rhMFG-E8 was purchased from R&D Systems, Inc. (McKinley, NE, USA) and injected at 1 h after TBI induction, doses of rhMFG-E8 were determined according to previous study^18,23^. LY294002 (Sigma-Aldrich, USA, 5 and 20 µM) was dissolved in a sterile saline solution containing 1% dimethyl sulfoxide (DMSO) and injected at 1 h before TBI induction. According to the detection of siRNA effect and previous study^18^, MFG-E8 siRNA, integrin β3 siRNA, and scramble siRNA (500 pmol/3 µl, Santa Cruz Biotechnology) were injected into the right lateral ventricles at a rate of 0.5 µl/min with a 10-µl Hamilton syringe at 2 days before TBI induction.

### Immunofluorescence staining

Immunofluorescence staining was performed as previously described^24^. Briefly, the immunostaining protocol was as follows: rats were deeply euthanized and perfused with 4% paraformaldehyde in 0.1 mM phosphate-buffered saline (PBS, pH 7.4). Brain samples were immersed in 30% sucrose until sinking to the bottom. 8 µm-thick slices were cut with a cryostat. The slices of each coronal sections were incubated in blocking buffer for 2 h, then washed with PBS three times for 10 min. Next, the slices were incubated with anti-MFG-E8 (1:200) and anti-cleaved caspase-3 antibody (1:100) respectively, in a dark place overnight at 4 °C. Afterwards, the slices were washed three times with PBS and incubated with another antibody, namely anti-NeuN (1:100), anti-GFAP (1:100), anti-Iba-1 (1:50), under similar conditions. The following day, the slices were thoroughly washed with PBS and incubated with the corresponding secondary antibodies for 1 h. After the wash with PBS, the slices were stained with DAPI for 2 min to show the location of the nucleus. Coverslips were applied with mounting media. The fluorescently-stained cells were imaged on an Olympus IX71 inverted microscope system and analyzed using the Image-Pro Plus 6.0 software (Media Cybernetics, Silver Spring, MD, USA). All of the processes were conducted by two investigators who were blinded to the grouping.

### Western blot analysis

Western blot analysis was performed as previously described^25,26^. Briefly, the total protein concentration of the lysate was determined by the Bradford method using the Bradford Protein Assay Kit (Beyotime Biotechnology, Shanghai, China). Equal amounts of proteins were resolved on a 10–12% sodium dodecyl sulfate-polyacrylamide gel electrophoresis (SDS-PAGE) gel and transferred onto polyvinylidene fluoride (PVDF) membrane (Immobilon-P, Millipore, Billerica, MA, USA). The membrane was blocked with 5% non-fat dry milk in TBST (Tris-buffered saline with 0.05% Tween 20) for 2 h at room temperature, and then incubated overnight at 4 °C, separately with the appropriate primary antibodies against the specific proteins. Specifically, MFG-E8 (Santa Cruz Biotechnology, USA, 1:200), AQP4 (Santa Cruz Biotechnology, USA, 1:200), Integrin β3 (Santa Cruz Biotechnology, USA, 1:200), p-FAK (Cell Signaling Technology, USA, 1:1000), FAK (Cell Signaling Technology, USA, 1:1000), p-AKT (Cell Signaling Technology, USA, 1:1000), AKT (Cell Signaling Technology, USA, 1:1000), caspase-3 (Cell Signaling Technology, USA, 1:1000), Bcl-2 (Cell Signaling Technology, USA, 1:1000), Bax (Abcam, Cambridge, UK, 1:200), and β-actin (Bioworld Technology, USA, 1:5000) in a blocking buffer. Afterwards, the membrane was washed three times with TBST for 15 min, and then incubated with the secondary antibodies, namely HRP conjugated secondary antibodies (goat; Bioworld Technology, USA, 1:5000) or HRP conjugated secondary antibodies (horse; Cell Signaling Technology, USA, 1:1000) for 2 h at room temperature. Finally, following a 20-min wash with TBST, the protein bands were visualized via enhanced chemiluminescence (ECL) (Millipore, Billerica, MA, USA) and exposure to X-ray film. The western blot results were analyzed using Un-Scan-It 6.1 software (Silk Scientific Inc., Orem, UT, USA).

### Quantitative real-time polymerase chain reaction

Total RNA was extracted from tissues using Trizol reagent (Invitrogen Life Technologies, Carlsbad, CA, USA). RNA quality was insured by gel visualization and spectrophotometric analysis (OD260/280). After reverse transcription, quantitative analysis of the MFG-E8, Integrin β3, IL-1β, IL-6, and IL-10 mRNA expression were performed with the real-time PCR method^24^ and the primers were synthesized by ShineGene Biotechnology (Shanghai, China) (Table 1). Test cDNA results were normalized to β-actin. All samples were analyzed in triplicate.

**Table 1:** Polymerase chain reaction (PCR) primer sequences

Target gene	Forward (5′ to 3′)	Reverse (5′ to 3′)
MFG-E8	CGGGCCAAGACAATGACATC	TCTCTCAGTCTCATTGCACACAAG
Integrin β3	CGTCAGCCTTTACCAGAATTATAGTG	TTTCCCGTAAGCATCAACAATG
β-Actin	CGTGAAAAGATGACCCAGATCA	CACAGCCTGGATGGCTACGTA
IL-1β	AAGCCTCGTGCTGTCGGACC	TGAGGCCCAAGGCCACAGG
IL-6	GAGACTTCCATCCAGTTGCCT	TGGGAGTGGTATCCTCTGTGA
IL-10	GGTTGCCAAGCCTTATCG A	ACCTGCTCCACTGCCTTGCT

### Terminal deoxynucleotidyl transferase dUTP nick end-labeling (TUNEL) staining

The brain tissue sections were examined for apoptotic cells using a terminal deoxynucleotidyl transferase dUTP nick end-labeling (TUNEL) detection kit (Roche, South San Francisco, CA, USA) following the manufacturer’s instructions. Briefly, the sections were incubated with the TUNEL reagent for 1 h at 37 °C. The sections were then washed three times in PBS, counterstained with DAPI for 2 min, and rinsed with PBS. Coverslips were applied with mounting media. Fluorescence was imaged on an Olympus IX71 inverted microscope system. The number of apoptotic cells to DAPI stained-cells was regarded as apoptotic index (apoptotic cells/DAPI). Six random vision fields (40×) surrounding contusion in each coronal section were chosen, and the mean number of apoptotic index in the six views was regarded as the data of each section. A total of four sections from each animal were used for quantification. The final average number of the four sections was regarded as the data for each sample. Data are presented as the mean number of apoptotic index per 40× magnification field. All the processes were conducted by two investigators who were blinded to the grouping.

### Fluoro-Jade C (FJC) staining

FJC staining (Merckmillipore, Germany) was performed according to the operation instructions and to detect degenerating neurons. Briefly, frozen sections were prepared, fixed, and immersed in a basic alcohol solution consisting of 1% sodium hydroxide in 80% ethanol for 5 min, then rinsed for 2 min each in 70% ethanol and distilled water, and then incubated in 0.06% potassium permanganate solution for 10 min. Following a 1–2 min water rinse, the slices were transferred for 10 min to a 0.0001% solution of FJC dissolved in 0.1% acetic acid vehicle and then rinsed through three changes of distilled water for 1 min per change. The slices were air-dried, coverslips were applied, and the sections were visualized on an Image J software (Image J 1.4, NIH, USA). Two observers blinded to the experimental group counted the FJC-positive cells in six sections per brain (at 40× magnification) through the injury’s epicenter. The data were presented by the average number of FJC-positive neurons in the fields.

### Brain water content

Brain water content was measured as previously studied^27^. Animals were anesthetized with sodium pentobarbital (50 mg/kg; i.p.) and the brains were quickly dissected at 24 and 72 h after TBI. The brainstem was discarded, while the tissue of ipsilateral cortex, the contralateral cortex, and the cerebellum were harvested. The wet weight of each cortical tissue was measured, then dried for 72 h at 80 °C and the dry weight determined. All experiments were conducted by two investigators who were blinded to the grouping. The percentage of brain water content was calculated using the following formula: =[(wet weight−dry weight)/wet weight] × 100%.

### Neurologic scoring

The neurological function of all rats before TBI and at 24 and 72 h after TBI were evaluated, according to previous studies^28,29^, by three investigators who were blinded to the experimental groups. The grading of neurological severity scoring (NSS) was as follows: severe injury (score: 13–18); moderate injury (score: 7–12); mild injury (score: 1–6); no injury (score: 0) (Table 2).

**Table 2:** Neurological severity scores (NSS)

	Points
**Motor tests**	
Raising rat by the tail (normal = 0; maximum = 3)	
Flexion of forelimb	1
Flexion of hindlimb	1
Head moved >10° to vertical axis within 30 s	1
Placing rat on the floor (normal = 0; maximum = 3)	
Normal walk	0
Inability to walk straight	1
Circling toward the paretic side	2
Fall down to the paretic side	3
**Sensory tests** (normal = 0; maximum = 2)	
Placing test (visual and tactile test)	1
Proprioceptive test (deep sensation, pushing the paw against the table edge to stimulate limb muscles)	2
**Beam balance tests** (normal = 0; maximum = 6)	
Balances with steady posture	0
Grasps side of beam	1
Hugs the beam and one limb falls down from the beam	2
Hugs the beam and two limbs fall down from the beam, or spins on beam (>60 s)	3
Attempts to balance on the beam but falls off (>40 s)	4
Attempts to balance on the beam but falls off (>20 s)	5
Falls off: no attempt to balance or hang on to the beam (<20 s)	6
**Reflexes absent and abnormal movements** (normal = 0; maximum = 4)	
Pinna reflex (head shake when touching the auditory meatus)	1
Corneal reflex (eye blink when lightly touching the cornea with cotton)	1
Startle reflex (motor response to a brief noise from snapping a clipboard paper)	1
Seizures, myoclonus, myodystony	1
Maximum points	18

### Statistical analysis

The SPSS 17.0 software package was used for the statistical analysis. All data are expressed as the mean ± standard deviation (SD). Comparisons between two groups were performed using Student’s *t* test and multiple comparisons were performed using a one-way ANOVA followed by Tukey’s test. A *P* value of <0.05 was regarded as statistically significant.

## Results

### General observations and mortality rate

**Fig. 2:**
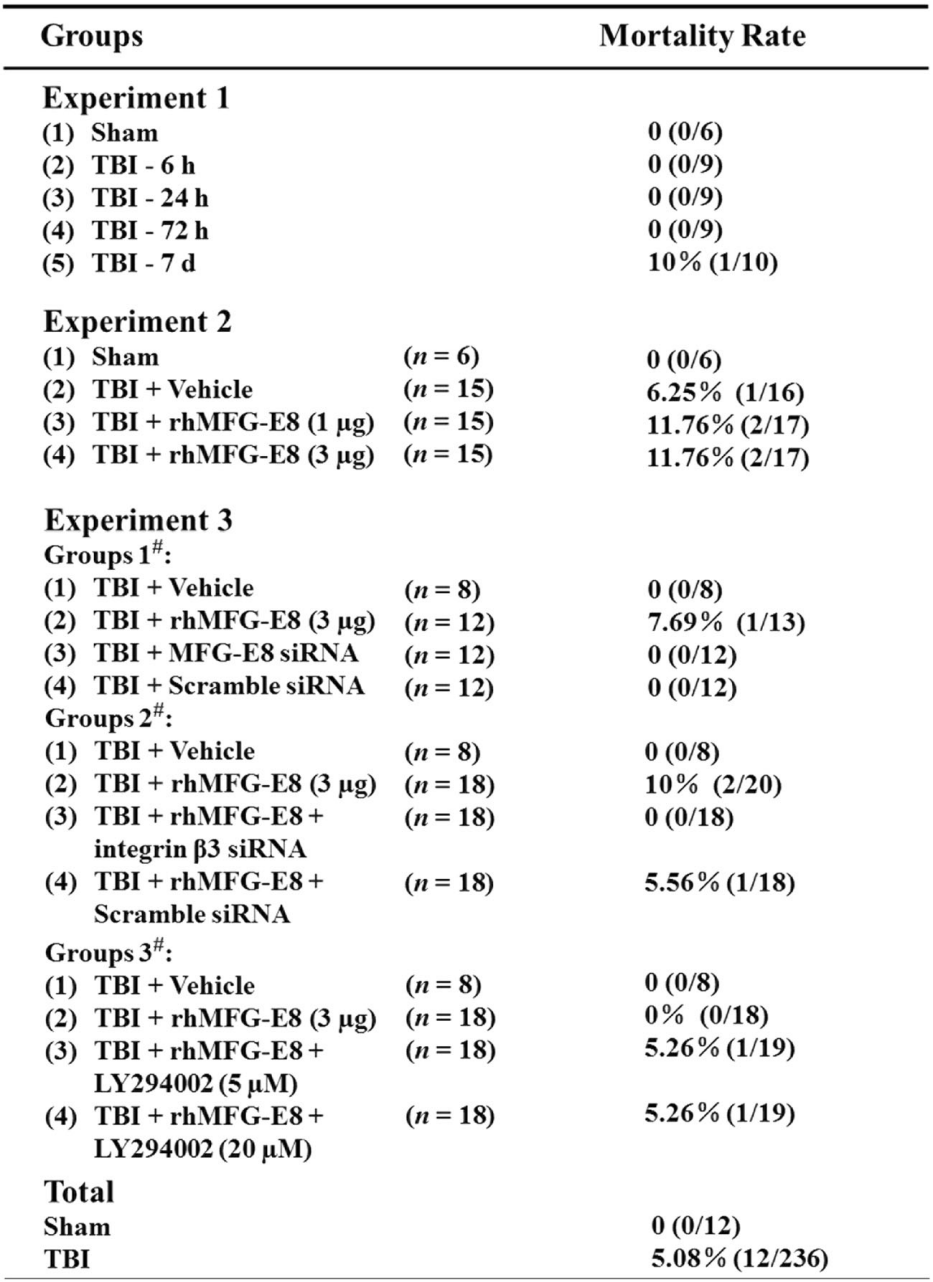
Mortality rate and general observations

Out of the 236 surgeries in rats that were performed, 12 rats died due to overdose anesthesia or serious injury in the TBI group. No rats died in Sham group 0% (0/12), and the overall mortality rate of TBI in rats was 5.08% (Fig. 2). There was no statistical difference in body weight and body temperature in any of the experimental groups (data not shown).

### Increased MFG-E8 expression peaks 24 h after TBI and is mainly expressed in microglia

**Fig. 3:**
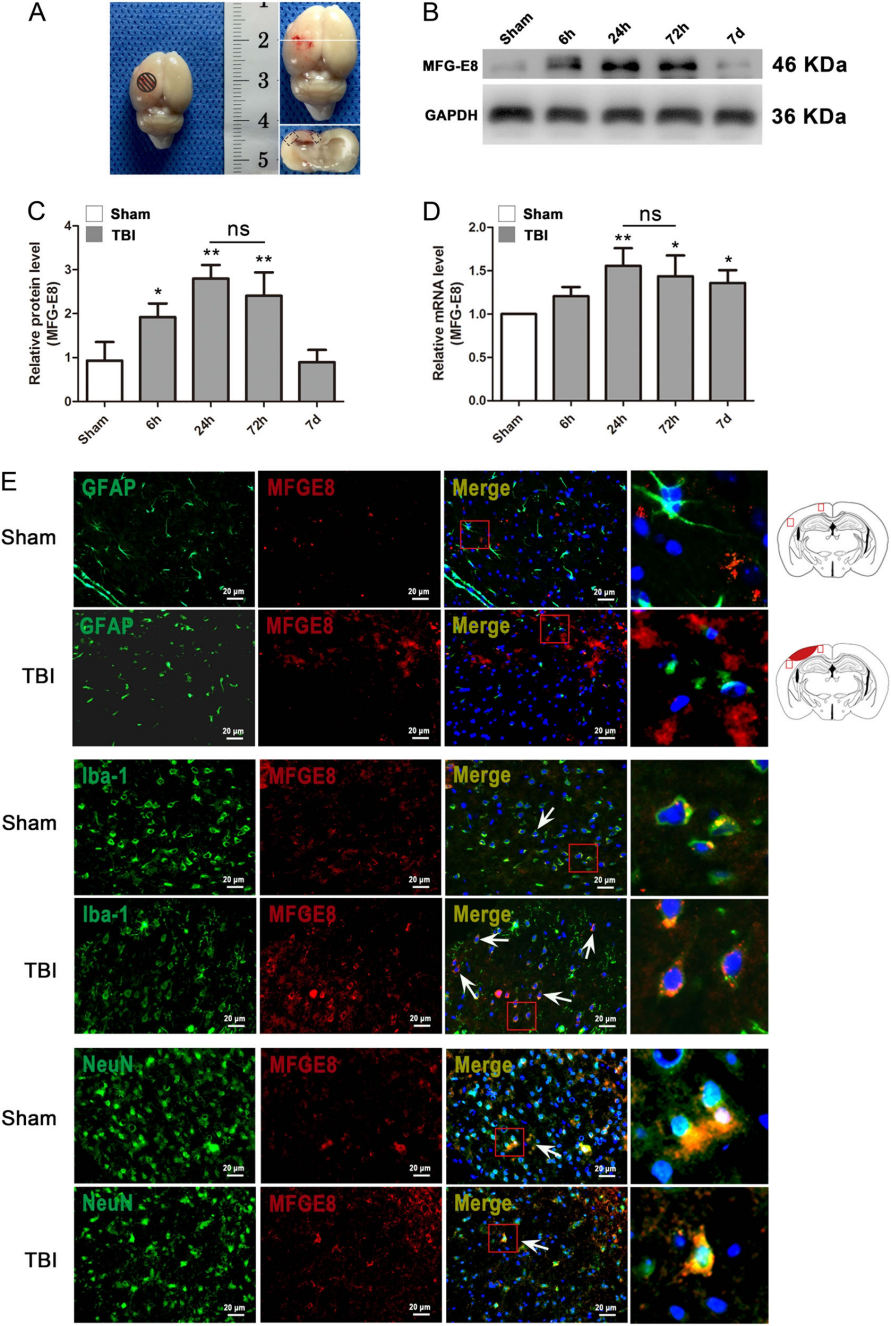
The expression of MFG-E8 on the mRNA and protein level and its cellular distribution after TBI. Experimental TBI model in SD rats and the tissue around the lesion in cortex was sampled for western blot and quantitative real-time PCR analysis in this study (**a**). The mRNA and protein expressions of MFG-E8 were measured at 6 h, 24 h, 72 h, and 7 days after TBI using western blotting and quantitative real-time PCR (**b**). The mRNA and protein expressions of MFG-E8 were significantly increased at 24 and 72 h after TBI as compared with the Sham group (**c**, **d**). Representative immunofluorescence co-staining images of MFG-E8/GFAP, MFG-E8/Iba-1, MFG-E8/NeuN in the Sham group and TBI group (24 h). Astrocytes, microglial cells, and neuron cells are labeled with GFAP (green), Iba-1 (green), and NeuN (green), respectively. (MFG-E8 = red, DAPI = blue). Diagram of coronal rat brain section showing the location of lesion cavity (red) and photograph region (red squares). The quantitative data are the mean ± SD (*n* = 9 each; *P < 0.05; **P < 0.01 vs. Sham group; ^ns^*P* > 0.05). Bar = 20 µm

To determine the role of MFG-E8 in rats after TBI, we first studied the expression and cellular localization of MFG-E8 in left cerebral cortical tissues. MFG-E8 protein and gene expression were examined by western blot analysis and quantitative real-time PCR, respectively. Following TBI, MFG-E8 protein expression was significantly increased at the 6 h time point, reaching its highest levels at 24 h and lasting up to 7 days (Fig. 3b, c). mRNA expression showed a similar pattern. MFG-E8 mRNA levels in the TBI group at the 24 h time point increased 50% relative to the mRNA levels in the Sham group (Fig. 3d). To determine the cellular localization of MFG-E8 in damaged cortical tissues, we conducted double-immunofluorescence staining. MFG-E8 was found to locate exclusively to the cytoplasm and was primarily expressed in microglial cells with minimal expression in neurons. MFG-E8 was rarely expressed in astrocytes.

### rhMFG-E8 decreased brain water content and improved neurological function after TBI in rats

**Fig. 4:**
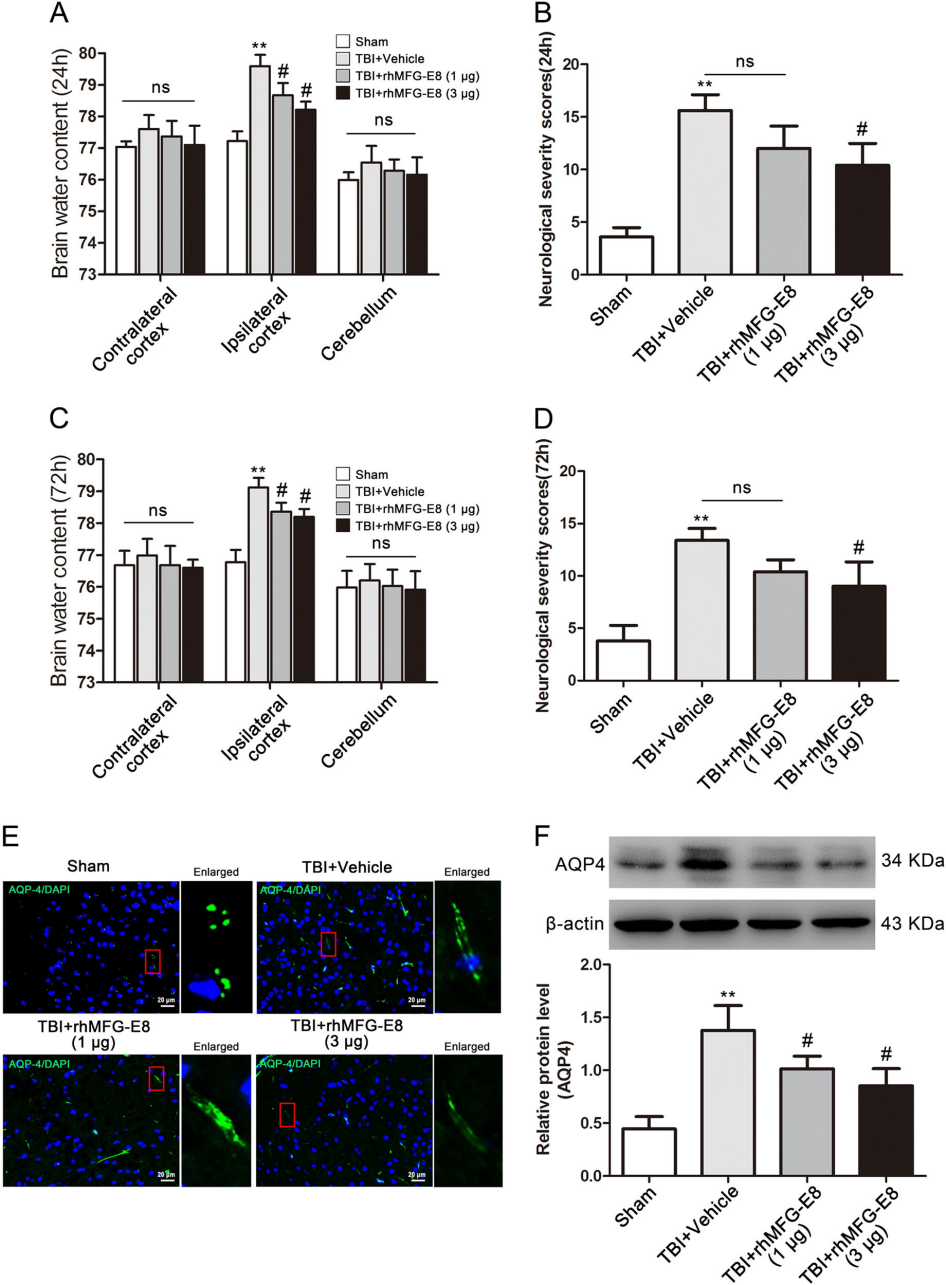
The effect of rhMFG-E8 on neuroprotection was evaluated by brain water content, neurological severity scores at 24 and 72 h after TBI. Both two dosages of rhMFG-E8 decreased brain edema in the ipsilateral cortex at 24 and 72 h after TBI (**a**, **c**), while only high dosage of rhMFG-E8 on neurological function has statistical differences compared with the TBI + Vehicle group at 24 and 72 h post-TBI (**b**, **d**). Representative immunofluorescence staining images of AQP4/DAPI (AQP4 = green, DAPI = blue) (**e**). Western blotting showed that administration of rhMFG-E8 decreased the expression of AQP-4 as compared with the TBI + Vehicle group at 24 h after TBI (**f**). The quantitative data are the mean ± SD (*n* = 15 each; **P < 0.01 vs. Sham group; ^#^*P* < 0.05 vs. TBI + Vehicle group; ^ns^*P* > 0.05). Bar = 20 µm

To investigate the neuroprotective effects of MFG-E8 after TBI in rats, two different dosages of rhMFG-E8 (1 and 3 μg) were administered after a TBI event. Brain water content and neurological severity scores were used to evaluate brain edema and neurological function, respectively. The results showed that for both dosages of rhMFG-E8, brain water content decreased in the ipsilateral cortex compared with the TBI + Vehicle control group at 24 and 72 h (Fig. 4a, c). Administration of 1 μg rhMFG-E8 did not improve the neurological deficits at 24 and 72 h, whereas 3 μg rhMFG-E8 significantly affected neurological function at 24 and 72 h post-TBI (Fig. 4b, d). Furthermore, the neurological severity scores in the TBI + rhMFG-E8 (3 μg) group decreased 33.33% relative to TBI + Vehicle group at 24 h and 32.83% at 72 h. This suggests treatment with 3 μg rhMFG-E8 at 24 h after TBI provides more effective neuroprotection. Accordingly, the high rhMFG-E8 dosage (3 μg) and most effective treatment time (24 h) were chosen for all subsequent mechanistic experiments, and the negative controls of rhMFG-E8 were also provided (Supplementary Figure 1).

The effect of rhMFG-E8 on AQP4 expression, a protein that plays a key role in the brain edema process, was also assessed after TBI through western blot analysis and immunofluorescence staining. AQP4 protein levels were significantly increased in the TBI + Vehicle group compared with the Sham group, while rhMFG-E8 treatment reversed AQP4 expression, with a high rhMFG-E8 dosage being more effective (Fig. 4e, f).

### Administration of rhMFG-E8 reduces neuronal apoptosis at 24 h after TBI in rats

**Fig. 5:**
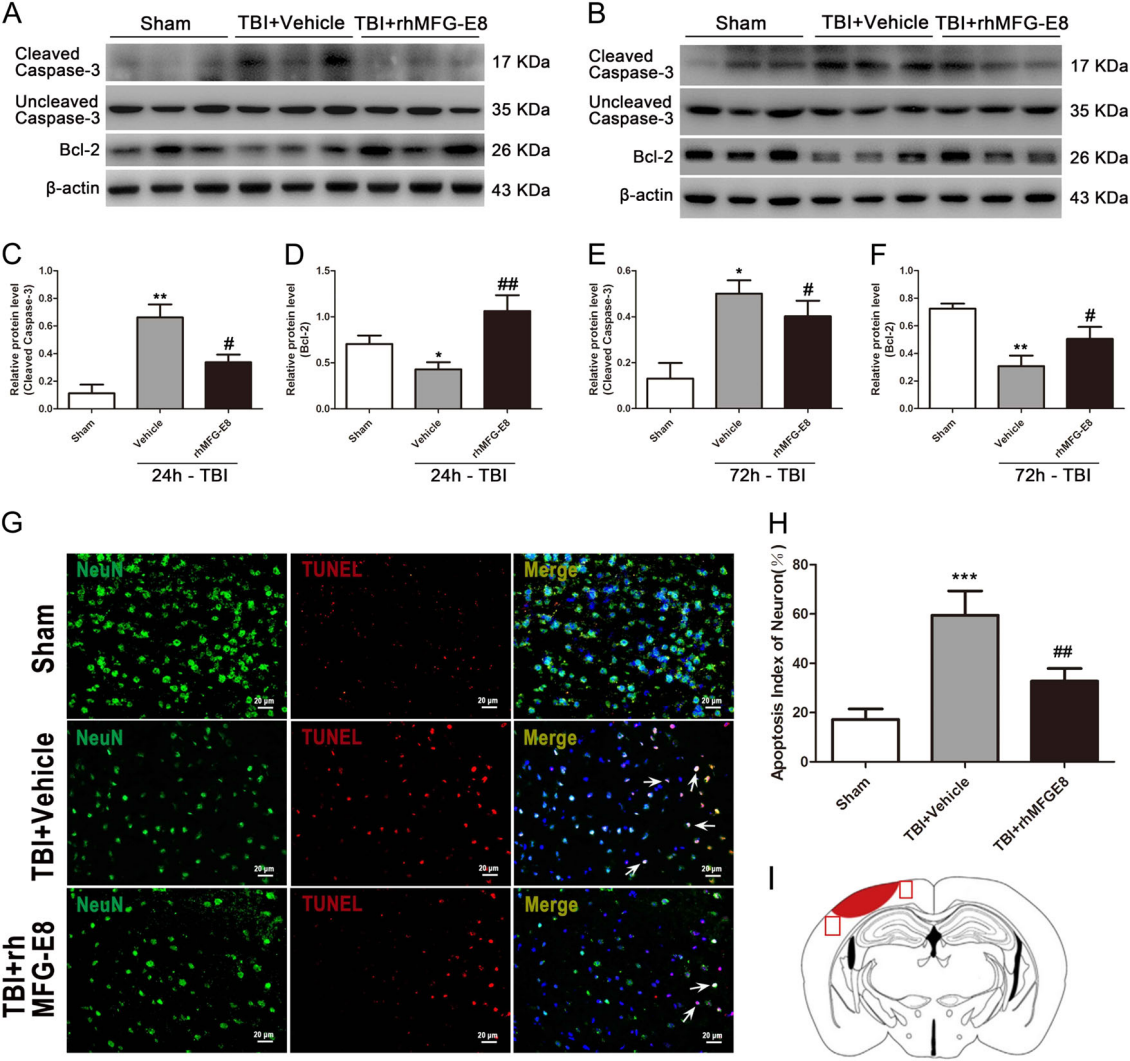
The effect of rhMFG-E8 on neuronal apoptosis was evaluated by western blotting and immunofluorescence co-staining at 24 and 72 h after TBI. Western blotting showed that rhMFG-E8 (3 μg) administration decreased the expression of cleaved caspase-3 and increased the expression of Bcl-2 at 24 and 72 h after TBI (**a**–**f**). Representative immunofluorescence staining images of NeuN/DAPI (NeuN = green, TUNEL = red) (**g**), results showed administration of rhMFG-E8 decreased the amount of TUNEL-positive neurons at 24 h after TBI (**h**). Diagram of coronal rat brain section showing the location of lesion cavity (red) and photograph region (red squares) (**i**). The quantitative data are the mean ± SD (*n* = 15 each; *P < 0.05, **P < 0.01 vs. Sham group; **P < 0.01 vs. TBI + Vehicle group; ^#^*P* < 0.05, ^##^*P* < 0.01 vs. TBI + Vehicle group). Bar = 20 µm

Neuronal apoptosis plays an important role in the pathogenesis of the secondary insult following TBI. To investigate the potential role of MFG-E8 after TBI in rats, we first examined the protein levels of the apoptosis indicators, cleaved caspase-3 and Bcl-2, via western blot analysis. Following TBI, the levels of the pro-apoptosis protein cleaved caspase-3 were significantly increased, while the anti-apoptosis protein Bcl-2 was markedly decreased in the TBI + Vehicle group compared with the Sham group at both 24 and 72 h (Fig. 5a–f). By contrast, the expression of cleaved caspase-3 and Bcl-2 were reversed in TBI + rhMFG-E8 group relative to the TBI + Vehicle group. Treatment with rhMFG-E8 at 24 h after TBI also provided a more marked effect than at 72 h (Fig. 5a–f).

TUNEL immunofluorescence staining was also used to evaluate the role of rhMFG-E8 in anti-apoptosis after TBI. The results showed that the number of TUNEL-positive neurons in TBI + Vehicle group were significantly increased compared to the Sham group, while administration of rhMFG-E8 reduced the amount of TUNEL-positive neurons at 24 h post-TBI (Fig. 5g, h).

**Fig. 6:**
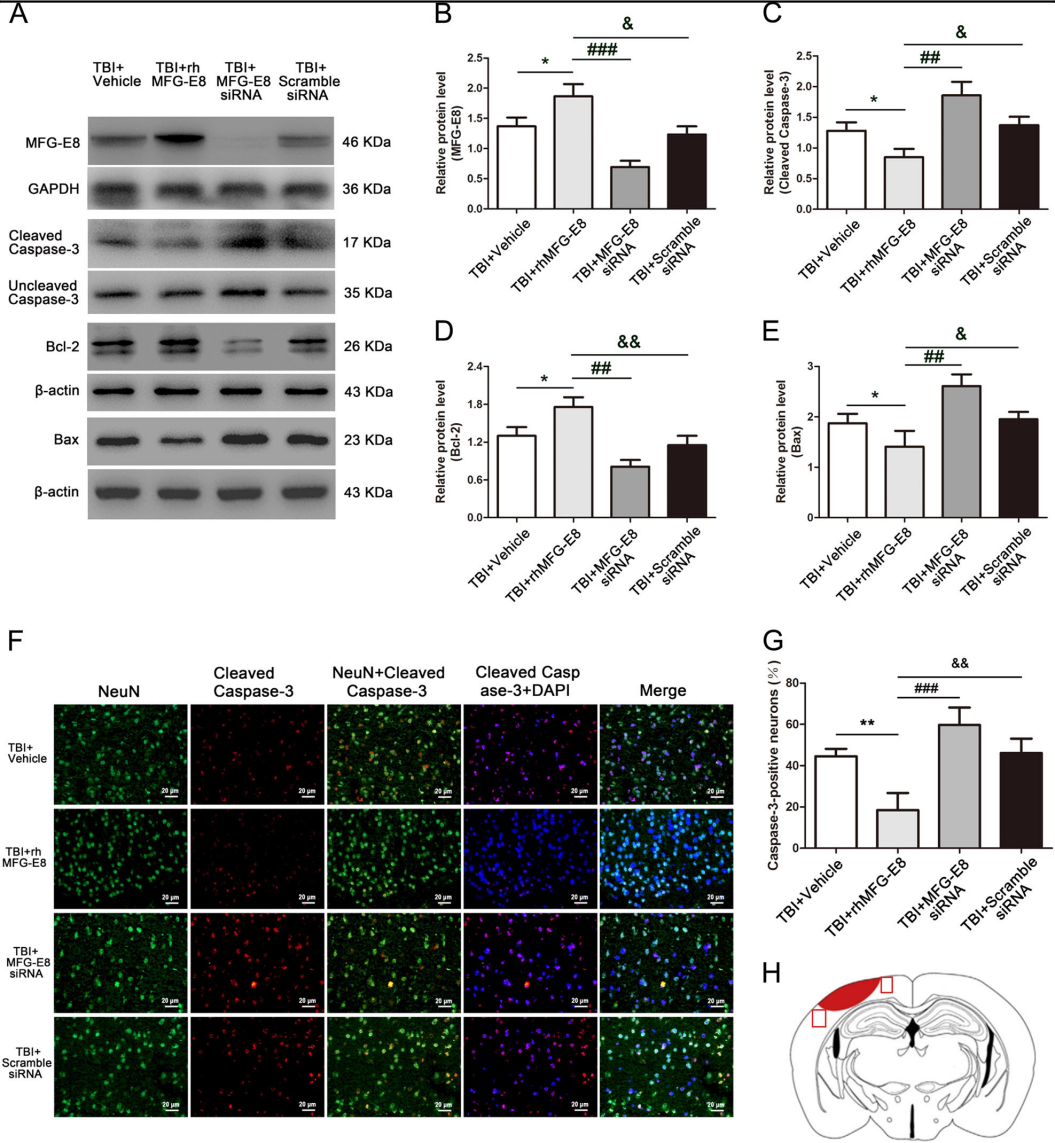
Administration of MFG-E8 siRNA, the role of rhMFG-E8 on anti-apoptosis was evaluated by western blotting and immunofluorescence co-staining at 24 h after TBI. Western blotting showed that rhMFG-E8 increased the levels of MFG-E8 and Bcl-2 (**a**, **b**, **d**), and decreased the expressions of cleaved caspase-3 and Bax (**a**, **c**, **e**) at 24 h after TBI. Representative immunofluorescence co-staining images of NeuN/Cleaved caspase-3 (NeuN = green, Cleaved caspase-3 = red, DAPI = blue) (**f**). The results showed treatment of rhMFG-E8 decreased the amount of Cleaved caspase-3-positive neurons, while MFG-E8 siRNA significantly increased the number of apoptotic neurons at 24 h after TBI (**g**). Diagram of coronal rat brain section showing the location of lesion cavity (red) and photograph region (red squares) (**h**). The quantitative data are the mean ± SD (*n* = 12 each; *P < 0.05 vs. TBI + Vehicle group; ^##^*P* < 0.01, ^###^*P* < 0.001 vs. TBI + rhMFG-E8 group; ^&^*P* < 0.05, ^&&^*P* < 0.01 vs. TBI + rhMFG-E8 group). Bar = 20 µm

To further verify the anti-apoptosis effect of rhMFG-E8, we administrated MFG-E8 siRNA treatment. Treatment with rhMFG-E8 markedly increased MFG-E8 protein expression compared with the TBI + Vehicle group, while MFG-E8 siRNA treatment significantly decreased MFG-E8 protein levels at 24 h following TBI (Fig. 6a, b). Similarly, the levels of pro-apoptosis-associated proteins in TBI + rhMFG-E8 group, such as cleaved caspase-3 and Bax, were significantly down-regulated relative to the TBI + Vehicle group, while the expression of pro-survival protein Bcl-2 was up-regulated (Fig. 6a, c–e). After treatment with MFG-E8 siRNA, the levels of apoptosis-associated proteins were reversed compared with the TBI + rhMFG-E8 group (Fig. 6a, c–e). We also used double-immunofluorescence staining to evaluate the role of rhMFG-E8 on neuronal apoptosis. The number of cleaved caspase-3 positive-neurons in TBI + rhMFG-E8 group were significantly decreased relative to the TBI + Vehicle group, while they were increased after MFG-E8 siRNA treatment (Fig. 6f, g).

### Integrin-β3 knockdown and PI3K inhibition abolish the anti-apoptosis effect of rhMFG-E8 at 24 h after TBI

**Fig. 7:**
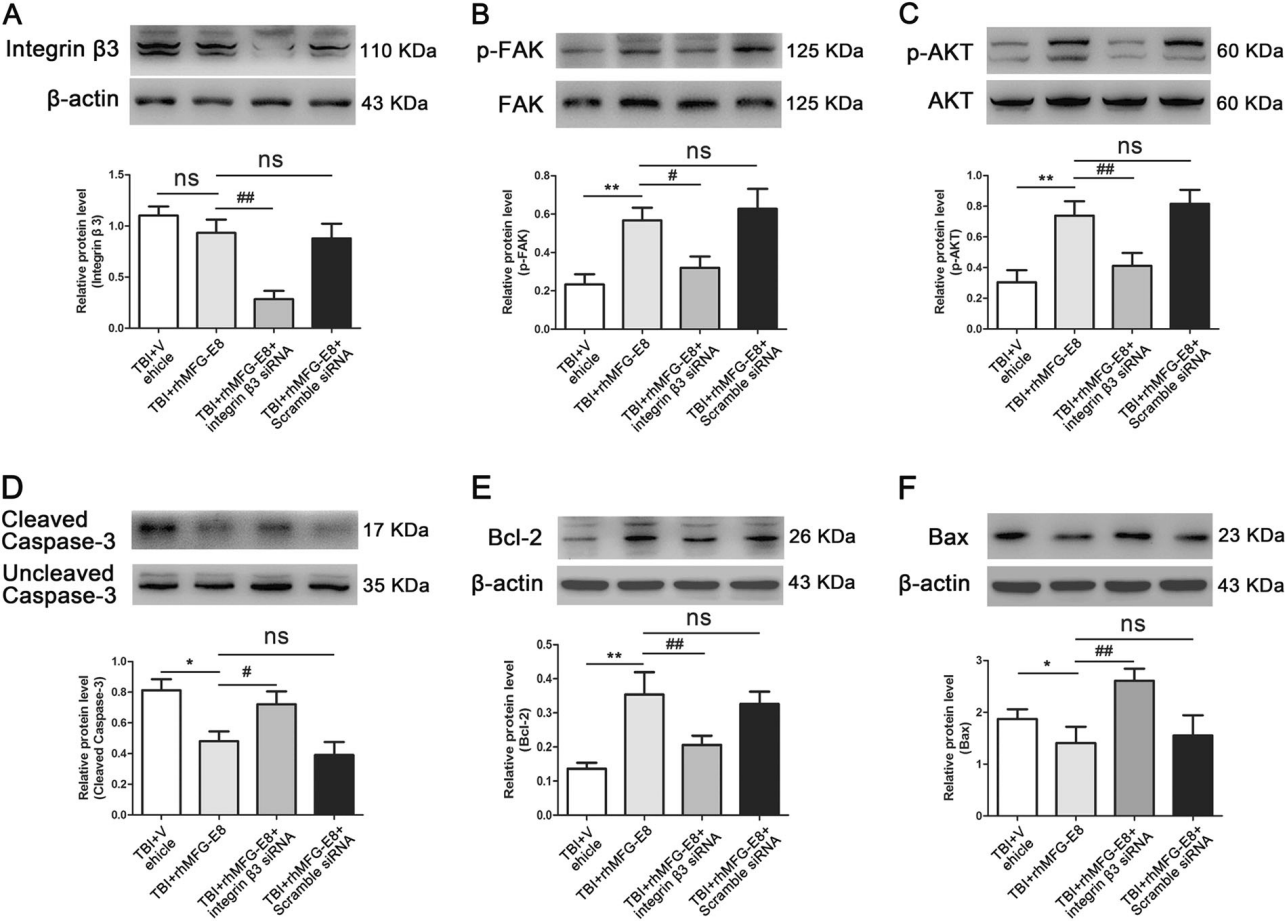
The effect of integrin β3 on rhMFG-E8's anti-apoptosis was evaluated by western blotting at 24 h after TBI. Western blotting showed that administration of rhMFG-E8 increased the expressions p-FAK and p-AKT, which were the downstream factors of integrin β3 receptor (**b**, **c**), while had no effect on the level of integrin β3 receptor (**a**). Treatment with integrin β3 siRNA, the levels of integrin β3 receptor, p-FAK and p-AKT were decreased, meanwhile reversed the role of rhMFG-E8 on anti-apoptosis (**d**–**f**). The quantitative data are the mean ± SD (*n* = 6 each; *P < 0.05, **P < 0.01 vs. TBI + Vehicle group; ^#^*P* < 0.05, ^##^*P* < 0.01 vs. TBI + rhMFG-E8 group; ^ns^*P* > 0.05)

To investigate the regulatory mechanism of rhMFG-E8 on apoptosis, integrin-β3 siRNA was used. The inhibitory effect of integrin-β3 siRNA was first examined via western blot analysis. Integrin-β3 expression showed no statistical difference between the TBI + Vehicle group and TBI + rhMFG-E8 group, while it was significantly decreased following treatment with integrin-β3 siRNA (Fig. 7a). Subsequently, the levels of integrin-β3/FAK/AKT pathway-associated proteins were assessed by western blot. The expression of p-FAK and p-AKT in the TBI + rhMFG-E8 group were markedly increased compared with the TBI + Vehicle group, while integrin-β3 siRNA treatment significantly inhibited the effect of rhMFG-E8 on p-FAK and p-AKT (Fig. 7b, c). In the TBI + rhMFG-E8 group, pro-survival protein Bcl-2 expression was up-regulated, whereas pro-apoptosis-associated protein cleaved caspase-3 and Bax were down-regulated. Treatment with integrin-β3 siRNA abolished these effects (Fig. 7d–f).

**Fig. 8:**
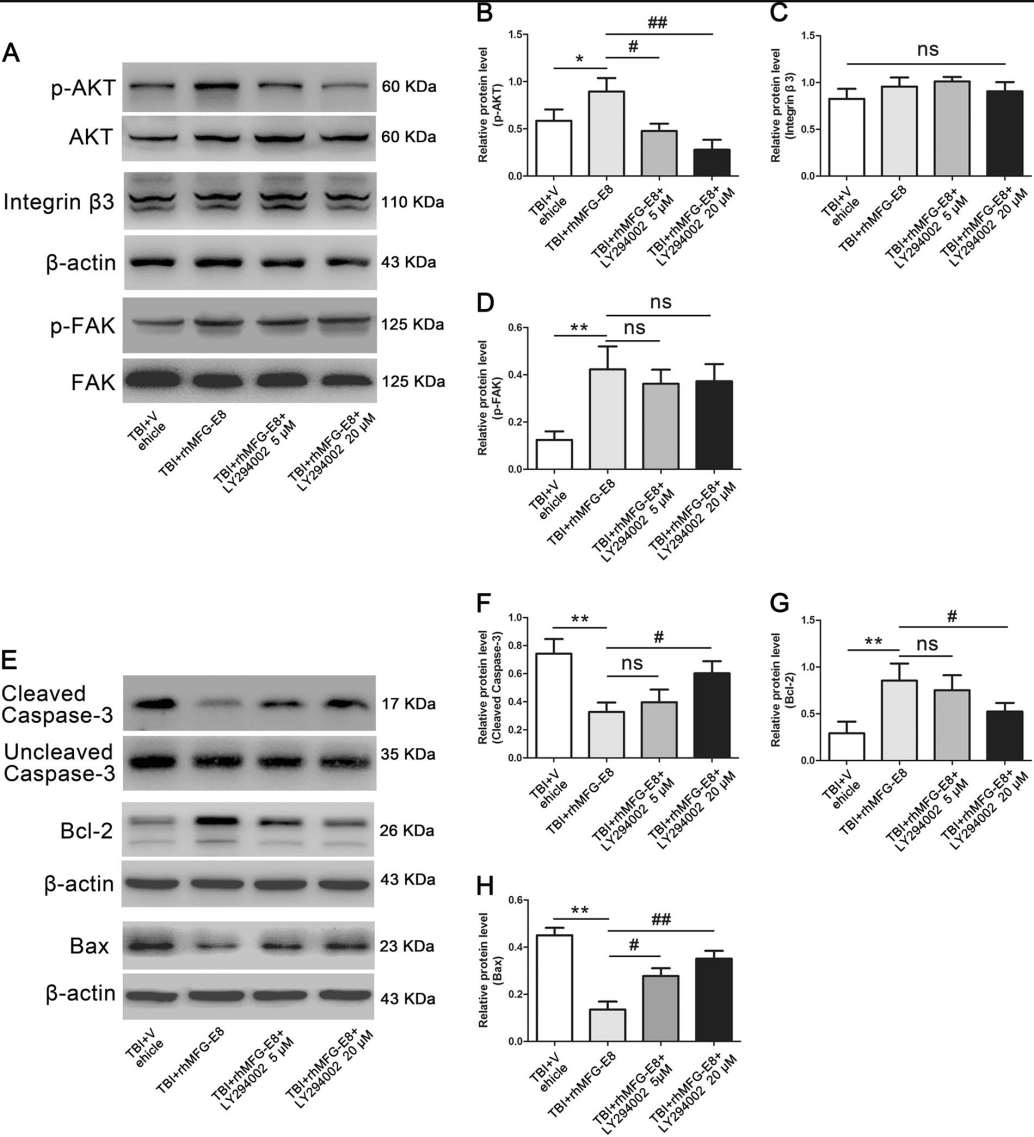
The role of integrin β3/FAK/PI3K/AKT signaling pathway on rhMFG-E8 of anti-apoptosis was evaluated by western blotting at 24 h after TBI. Western blotting showed that treatment with PI3K inhibitor, LY294002 (5 and 20 µM), decreased the expressions of p-AKT (**a**, **b**), high dosage exhibited better effect, while had no effect on integrin β3 and p-FAK (**a**, **c**, **d**). The expressions of pro-apoptosis-associated proteins, Cleaved caspase-3, and Bax were increased after administration of LY294002 (**e**, **f**, **h**), while the level of anti-apoptosis protein Bcl-2 was decreased (**e**, **g**). The quantitative data are the mean ± SD (*n* = 6 each; *P < 0.05, **P < 0.01 vs. TBI + Vehicle group; ^#^*P* < 0.05, ^##^*P* < 0.01 vs. TBI + rhMFG-E8 group; ^ns^*P* > 0.05)

**Fig. 9:**
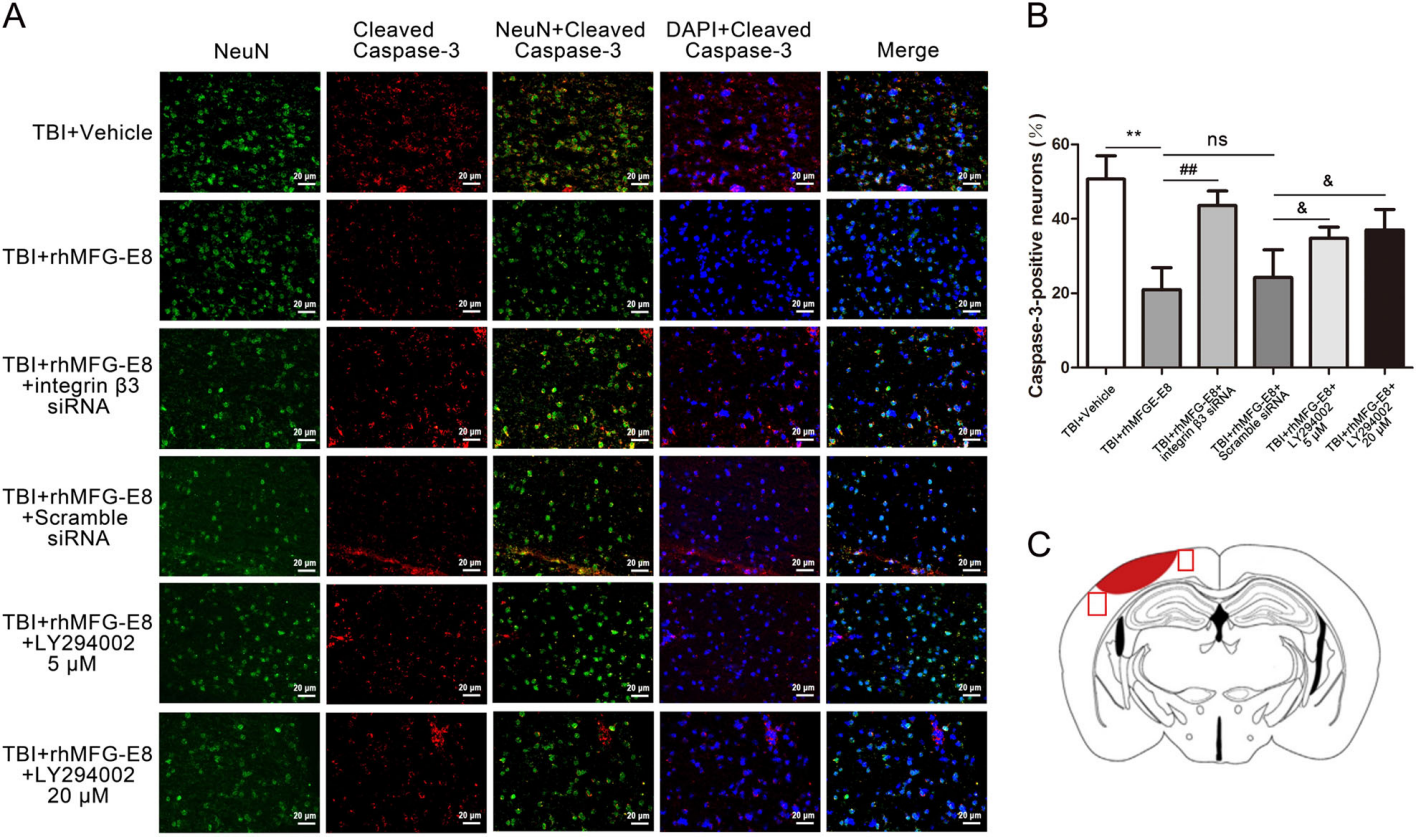
Inhibited the integrin β3/FAK/PI3K/AKT signaling pathway, the role of rhMFG-E8 on anti-apoptosis was evaluated by immunofluorescence co-staining at 24 h after TBI. Representative immunofluorescence co-staining images of Cleaved Caspase-3/NeuN, (Cleaved caspase-3 = red, NeuN = green, DAPI = blue) (**a**). The results showed treatment of rhMFG-E8 decreased the amount of Cleaved caspase-3-positive neurons, while Integrin β3 siRNA and LY294002 (5 and 20 µM) significantly reversed the role of rhMFG-E8 on anti-apoptosis at 24 h after TBI (**b**). Diagram of coronal rat brain section showing the location of lesion cavity (red) and photograph region (red squares) (**c**). Bar = 20 µm

Two dosages of PI3K inhibitor LY294002 (5 and 20 µM) were also evaluated. Western blot analysis revealed a significant decrease of p-AKT in the TBI + rhMFG-E8 + LY294002 group, with the higher dose of LY294002 providing a stronger effect (Fig. 8a, b). However, PI3K inhibitor LY294002 (5 and 20 µM) had no effect on the protein levels of integrin-β3 and p-FAK compared with the TBI + rhMFG-E8 group (Fig. 8a, c, d). In addition, the levels of cleaved caspase-3 and Bax were increased, while Bcl-2 decreased in the TBI + rhMFG-E8 + LY294002 group as compared with the TBI + rhMFG-E8 group. High doses of LY294002 exhibited a more pronounced effect on cleaved caspase-3, Bax, and Bcl-2 expression after rhMFG-E8 administration (Fig. 8e–h). Meanwhile, the percentage of cleaved caspase-3 positive-neurons in the TBI + rhMFG-E8 group were significantly decreased compared with the TBI + Vehicle group, whereas they were elevated by integrin-β3 siRNA or LY294002 (5 and 20 µM) treatment (Fig. 9a, b).

### MFG-E8 provides beneficial effects dependent on the integrin-β3/FAK/PI3K/AKT signaling pathway

**Fig. 10:**
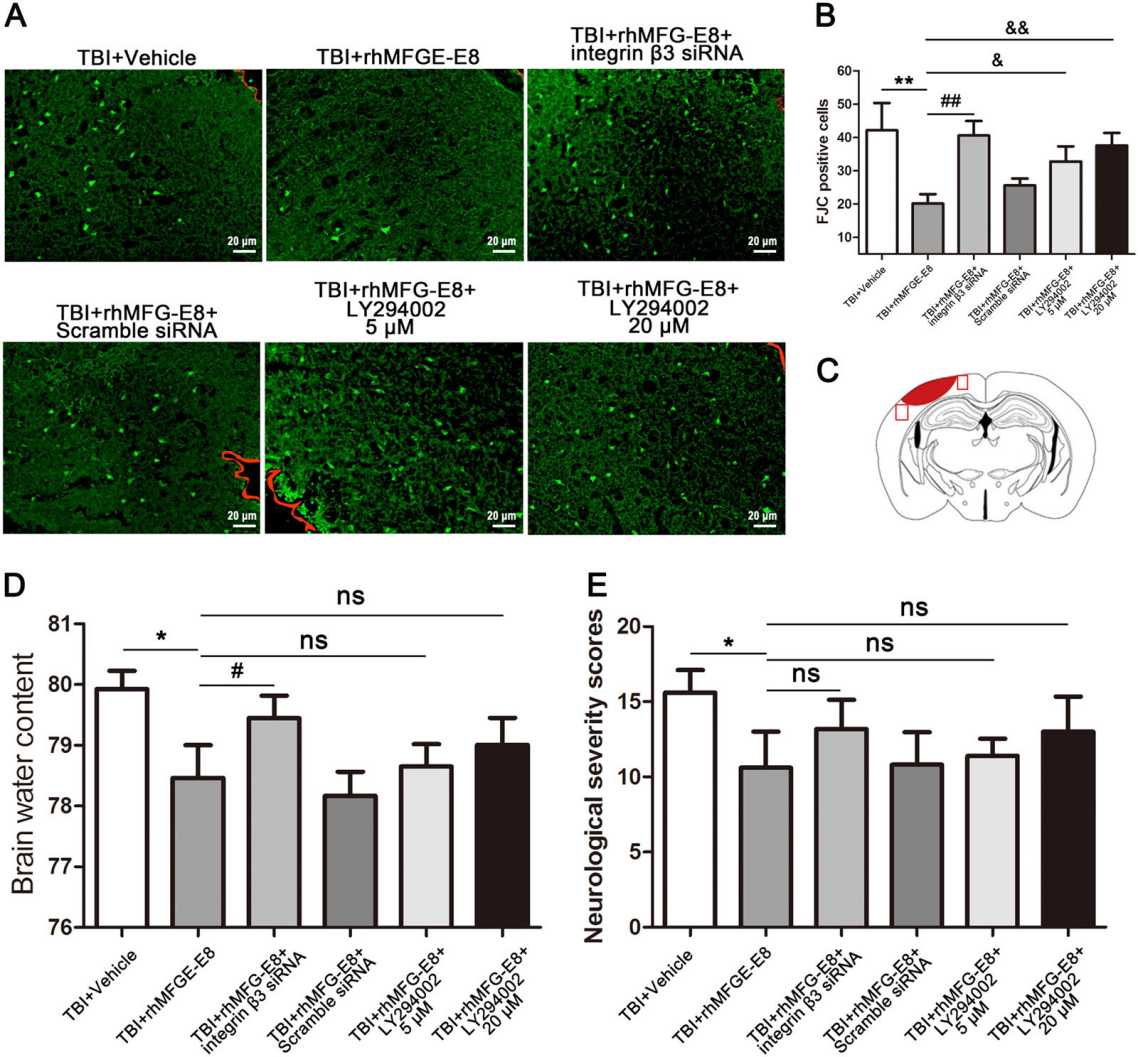
Inhibited the integrin β3/FAK/PI3K/AKT signaling pathway, the role of rhMFG-E8 on neuroprotection was evaluated by Fluoro-Jade C (FJC), brain water content, and neurological severity score at 24 h after TBI. Representative immunofluorescence staining images of FJC (green). The boundary of injury cortex was labeled by the red trajectory (**a**). The results showed treatment of rhMFG-E8 decreased the amount of FJC-positive cells, while increased it by Integrin β3 siRNA and LY294002 (5 and 20 µM) at 24 h after TBI (**b**). Diagram of coronal rat brain section showing the location of lesion cavity (red) and photograph region (red squares) (**c**). Administration of rhMFG-E8 decreased brain edema in the ipsilateral cortex, meanwhile decreased the neurological severity score at 24 h after TBI, while integrin β3 siRNA and LY294002 (5 and 20 µM) inverted the role of rhMFG-E8 on neuroprotection following TBI (**d**, **e**). The quantitative data are the mean ± SD (*n* = 6 each; *P < 0.05, **P < 0.01 vs. TBI + Vehicle group; ^#^*P* < 0.05, ^##^*P* < 0.01 vs. TBI + rhMFG-E8 group; ^ns^*P* > 0.05). Bar = 20 µm

Brain water content, neurological severity scores, and FJC staining were examined to evaluate the potential mechanisms of rhMFG-E8. We used integrin-β3 siRNA and PI3K inhibitor LY294002 (5 and 20 µM) to demonstrate whether the integrin-β3/FAK/PI3K/AKT signaling pathway participates in MFG-E8-induced neuroprotection. As shown in Fig. 10a, b, the number of FJC-positive cells in the ipsilateral cortex notably declined in the TBI + rhMFG-E8 group relative to the TBI + Vehicle group, whereas they increased in the TBI + rhMFG-E8 + integrin β3 siRNA and TBI + rhMFG-E8 + LY294002 (5 and 20 µM) groups. In addition, brain water content in the ipsilateral cortex and neurological severity scores decreased in the TBI + rhMFG-E8 (3 μg) group compared with the TBI + Vehicle group at 24 h after TBI, whereas they increased after administration of integrin β3 siRNA and PI3K inhibitor LY294002. However, no statistical difference was observed between the TBI + rhMFG-E8 and TBI + rhMFG-E8 + LY294002 (5 and 20 µM) groups (Fig. 10d, e).

## Discussion

This study presents the link between MFG-E8 and the integrin-β3/FAK/PI3K/AKT signaling pathway, both of which provide neuroprotection following TBI in rats. MFG-E8 was primarily located in microglia and neurons, and showed increased expression in the early stages of TBI. Treatment with rhMFG-E8 markedly alleviated TBI-induced brain edema and neurological deficits following TBI. Meanwhile, rhMFG-E8 treatment up-regulated the expression of integrin-β3/FAK/PI3K/AKT signaling pathway components, and decreased neuronal apoptosis after TBI. In contrast, integrin-β3 knockdown by siRNA and PI3K inhibitor LY294002 treatment abolished the effect of rhMFG-E8 on anti-apoptosis and neuroprotection. MFG-E8 is suggested to provide neuroprotection through modulation of the integrin-β3/FAK/PI3K/AKT signaling pathway, which may offer an attractive therapeutic target following TBI.

Increasing evidence points to the essential function of MFG-E8 in diverse physiological functions, including inflammation^5^, angiogenesis^30^, clearance of apoptotic cells^3,12^, and fertilization^7^. Several recent studies confirmed the therapeutic potential of MFG-E8 in various brain injury diseases, including neurodegenerative diseases^8,11,31^, cerebral ischemic injury^5,12^, and subarachnoid hemorrhage^18^. It has also been shown that MFG-E8 provides neuroprotection via suppressing inflammation and apoptosis, which may owe to the regulation of integrin-mediated cell adhesion and activation of the FAK-dependent phosphatidylinositol 3-kinase-AKT pathway^12,17^. Similarly, using an ischemic cerebral injury rat model that was binge-treated with the integrin-β3 receptor, MFG-E8 treatment reduced the infarct volume and accumulation of apoptotic cells, while simultaneously inhibiting the production of the inflammasome complex^5^. Liu et al. showed MFG-E8 was mainly expressed in macrophages and the levels of endogenous brain MFG-E8 increased in subarachnoid hemorrhage rats. In addition, exogenous rhMFG-E8 reduced brain edema and improved neurological function through attenuation of oxidative stress^18^. All of these studies indicate that MFG-E8 is essential for neuroprotection. Our results demonstrated that exogenous rhMFG-E8 could alleviate TBI-induced brain edema and neurological deficits, potentially through the inhibition of neuronal apoptosis, which was consistent with previous studies^18,32^. The effect of MFG-E8 on the reduction of brain edema might be through the modulation of the protein of AQP4. The previous study has shown that MFG-E8 could accelerate the tissue regeneration and neovascularization through modulation of VEGF-AKT signaling pathway^6^. Therefore, we supposed that the role of MFG-E8 on anti-brain edema might be bound up with VEGF-AKT pathway, not exclude the effect on the blood–brain barrier and its permeability. We will explore the mechanism of MFG-E8 on brain edema in the following experiments.

Apoptosis, as an important pathological process after TBI, can promote cell death through the triggering of devastating cascades^33^. Briefly, this owes to the reciprocal activation of apoptosis-related proteins (such as Bax, Bcl-2, and cleaved caspase-3) and cytoplasmic organelle damage, which lead to apoptosis^19^. Thereafter, apoptotic cells induce an immune response and tissue injury through the release of damage-associated molecular patterns (DAMPs)^21^. Meanwhile, previous studies have shown that the promotion of apoptotic cell removal was beneficial to various brain injury diseases^12,34,35^. In addition, MFG-E8 was shown to exhibit the physiological effect during cell–cell interaction through its highly conserved arginine–glycine–aspartate (RGD) motif, which could recognize the receptors of α_v_β_3/5_-integrin in macrophage cells^3^. While, under pathologic conditions, it has been reported that MFG-E8 could target the apoptotic cells by binding phosphatidylserine (PS) and induce apoptotic cell phagocytosis via the α_v_β_3/5_-integrin receptor of phagocytes^5,36^. Under neurodegenerative conditions, the MFG-E8 secreted from microglia could provide neuroprotection by enhancing oligomeric amyloid (oAβ) phagocytosis by microglia and reducing oAβ-induced neuronal cell death^11^. Whereas, the effect of MFG-E8 on TBI has not been previously investigated. In our study, we demonstrated that rhMFG-E8 inhibited neuronal apoptosis via α_v_β_3/5_-integrin receptor following TBI in rats, and, for the first time, we demonstrated the beneficial effect of rhMFG-E8 in TBI via regulation of apoptosis.

MFG-E8 modulated the expression of apoptosis-related proteins through its integrin α_v_β3 receptor, leading us to focus on the potential underlying mechanism. A previous study showed that the FAK-AKT survival pathway could be activated via the α_v_β3 integrin receptor and, through this pathway, elevated *bcl-2* gene transcription and the expression of anti-apoptotic protein Bcl-2^17^. Furthermore, the PI3K-AKT pathway has been confirmed to be neuroprotective through modulation of apoptosis in various brain injury diseases, including TBI^37,38^, neurodegenerative disease^39,40^, and cerebral ischemia^41,42^. We therefore speculated that the potential underlying mechanism of MFG-E8 on anti-apoptosis following TBI was through binding to the receptor of integrin-β3 and activating its downstream FAK/PI3K/AKT signaling pathway. For this purpose, we examined the expression of integrin-β3, FAK, and AKT. Treatment with rhMFG-E8 significantly increased p-FAK and p-AKT expression after TBI at 24 h, while integrin-β3 knockdown abolished the effect of rhMFG-E8 on anti-apoptosis and FAK/PI3K/AKT pathway activation. To further define the role of MFG-E8 on the FAK/PI3K/AKT signaling pathway, we also evaluated the PI3K kinase inhibitor LY294002, which displayed similar effects to that of integrin-β3 knockdown. The only difference was that LY294002 had no effect on the expression of integrin-β3 and p-FAK. We therefore concluded that MFG-E8 inhibited neuronal apoptosis and improved neurological function of TBI, which appears to occur through modulation of the integrin-β3/FAK/PI3K/AKT signaling pathway.

To the best of our knowledge, these results demonstrate for the first time the effect MFG-E8 has on integrin-β3/FAK/PI3K/AKT signaling, via the modulation of apoptosis after TBI. However, our study had several limitations. First, we only used MFG-E8 siRNA, integrin β3 siRNA, and LY294002 (PI3K kinase inhibitor), which might not have had an efficient inhibitor effect. Therefore, the data from gene knock-out rats (*mfg-e8*^−/−^ and *integrin* β*3*^−/−^) might be more persuasive. Second, we only examined the effect of MFG-E8 on integrin-β3/FAK/PI3K/AKT pathway, additional MFG-E8 signaling pathways affecting apoptosis should be studied further.

## Conclusion

MFG-E8 provides neuroprotection after TBI through inhibiting neuronal apoptosis, attenuating brain edema, and improving neurological function, while knocking down MFG-E8 and integrin β3 via siRNA, or inhibiting the activation of PI3K by LY294002 abolished the effect of rhMFG-E8 on anti-apoptosis following TBI. It is suggested that MFG-E8 reduced apoptosis might depend on integrin β3/FAK/PI3K/AKT pathway. Therefore, MFG-E8 might be a therapeutic target to improve neurological deficits and alleviate neuronal apoptosis after TBI.

## Electronic supplementary material

Supplementary material
