# Supplementary material for: Recombinant milk fat globule-EGF factor-8 reduces apoptosis via integrin β3/FAK/PI3K/AKT signaling pathway in rats after traumatic brain injury
Source: Cell Death Dis. 2018 Aug 28;9(9):845. doi: 10.1038/s41419-018-0939-5 (PMC6113274; doi:10.1038/s41419-018-0939-5)
Supplement: Supplementary file 1 — Supplementary material [file 41419_2018_939_MOESM1_ESM.docx]

**Supplementary Figures**


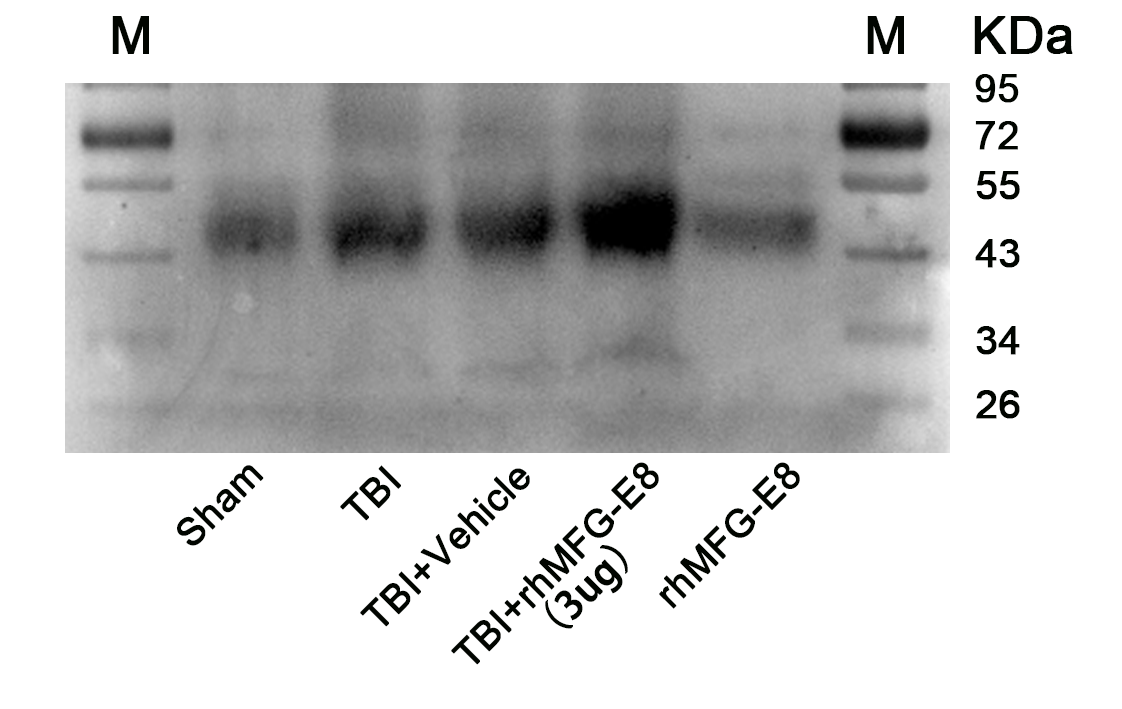


**Supplementary Figure 1.** Western blot showed the negative controls of rhMFG-E8.





**Supplementary Figure 2.** The effects of rhMFG-E8 on brain water content, neuronal apoptosis and integrin ꞵ3/PI3K/AKT signaling pathway were evaluated by western blotting at 24 h after TBI. Western blotting showed that treatment with rhMFG-E8 in Sham group had no effect on the levels of AQP4 (B), integrin ꞵ3 (D), Bax (E) and Cleaved caspase-3 (F) compared with the Sham group, while p-AKT (C) increased. The expression of AQP4, integrin ꞵ3, Bax and Cleaved caspase-3 were increased, meanwhile the p-AKT decreased after TBI, while administration of rhMFG-E8 reversed this expression. The quantitative data are the mean ± SD (n = 6, each; ^*^*P* ＜ 0.05, ^**^*P* ＜ 0.01 vs. Sham + rhMFG-E8 group; ^#^*P* ＜ 0.05 vs. TBI + Vehicle group; ^ns^*P* ＞ 0.05).


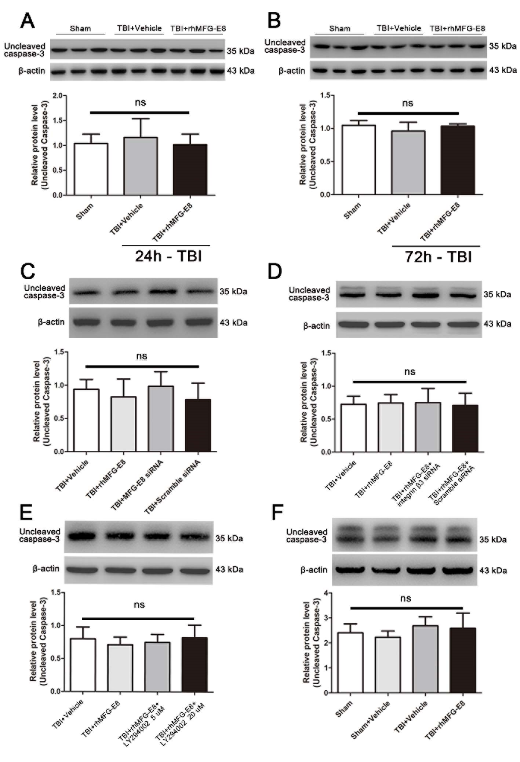


**Supplementary Figure 3.** The effect of rhMFG-E8 on apoptosis associated protein uncleaved caspase-3 was evaluated by Western blotting. The results showed that treatment with rhMFG-E8 (A, B, represent the Figure 5 as the supplementary data and F, represent the supplementary Figure 2), MFG-E8 siRNA (C, represent the Figure 6 as the supplementary data), integrin ꞵ3 siRNA (D, represent the Figure 7 as the supplementary data) and LY294002 (E, represent the Figure 8 as the supplementary data) had no effect on the level of uncleaved caspase-3 compared with the Sham group and TBI + Vehicle group. The quantitative data are the mean ± SD (n = 6, each; ^ns^*P* ＞ 0.05).


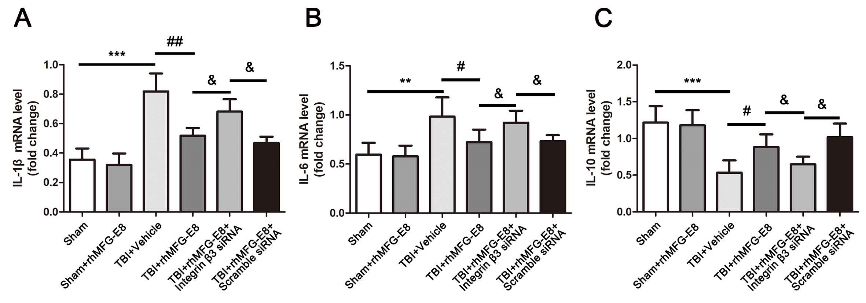


**Supplementary Figure 4**. The expression of neuro-inflammation related cytokines after treatment with integrin ꞵ3 siRNA were evaluated by Quantitative real-time PCR. The mRNA expression of IL-1ꞵ and IL-6 were significantly increased at 24 h after TBI as compared with the Sham group, while anti-inflammatory cytokine IL-10 was decreased. Administration of integrin ꞵ3 siRNA abolished the effect of rhMFG-E8 on anti-inflammation after TBI. The quantitative data are the mean ± SD (n = 6, each; ^**^*P* ＜ 0.01, ^***^*P* ＜ 0.001 vs. Sham group; ^#^*P* ＜ 0.05, ^##^*P* ＜ 0.01 vs. TBI + Vehicle group; ^&^*P* ＜ 0.05 vs. TBI + rhMFG-E8 group and TBI + rhMFG-E8 + integrin ꞵ3 siRNA group).





**Supplementary Figure 5**. The expressions of neuro-inflammation related cytokines after treatment with integrin ꞵ3 siRNA were evaluated by Western blotting. The protein levels of pro-inflammation factors IL-1ꞵ and IL-6 were dramatically increased, while anti-inflammatory cytokine IL-10 was decreased at 24 h after TBI as compared with the Sham group. Treatment with rhMFG-E8, the expressions of IL-1ꞵ and IL-6 were decreased, while the IL-10 was increased compared with TBI + Vehicle group. Administration of integrin ꞵ3 siRNA reversed the role of rhMFG-E8 on anti-inflammation after TBI. The quantitative data are the mean ± SD (n = 6, each; ^**^*P* ＜ 0.01 vs. Sham group; ^#^*P* ＜ 0.05 vs. TBI + Vehicle group; ^&^*P* ＜ 0.05 vs. TBI + rhMFG-E8 group and TBI + rhMFG-E8 + integrin ꞵ3 siRNA group).


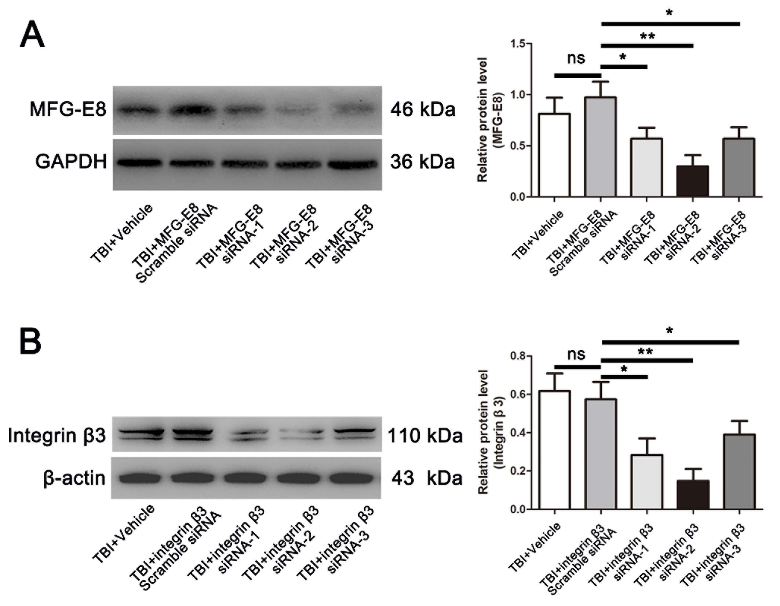


**Supplementary Figure 6**. The efficiency of three different sets of MFG-E8 siRNA and integrin ꞵ3 siRNA were used for knockdown MFG-E8 and integrin ꞵ3, which were evaluated by Western Blotting. MFG-E8 siRNA, integrin ꞵ3 siRNA and scramble siRNA (siRNA-1/100 pmol/3 μl, siRNA-2/500 pmol/3 μl, siRNA-3/1 nmol/3 μl) were injected into the right lateral ventricles at 2 d before TBI induction, which inhibited the expressions of MFG-E8 (siRNA-2) by 80％ and integrin ꞵ3 (siRNA-2) by 75％. The quantitative data are the mean ± SD (n = 4, each; ^*^*P* ＜ 0.05, ^**^*P* ＜ 0.01 vs. TBI + MFG-E8 scramble siRNA group and TBI + integrin ꞵ3 scramble siRNA group; ^ns^*P* ＞ 0.05).


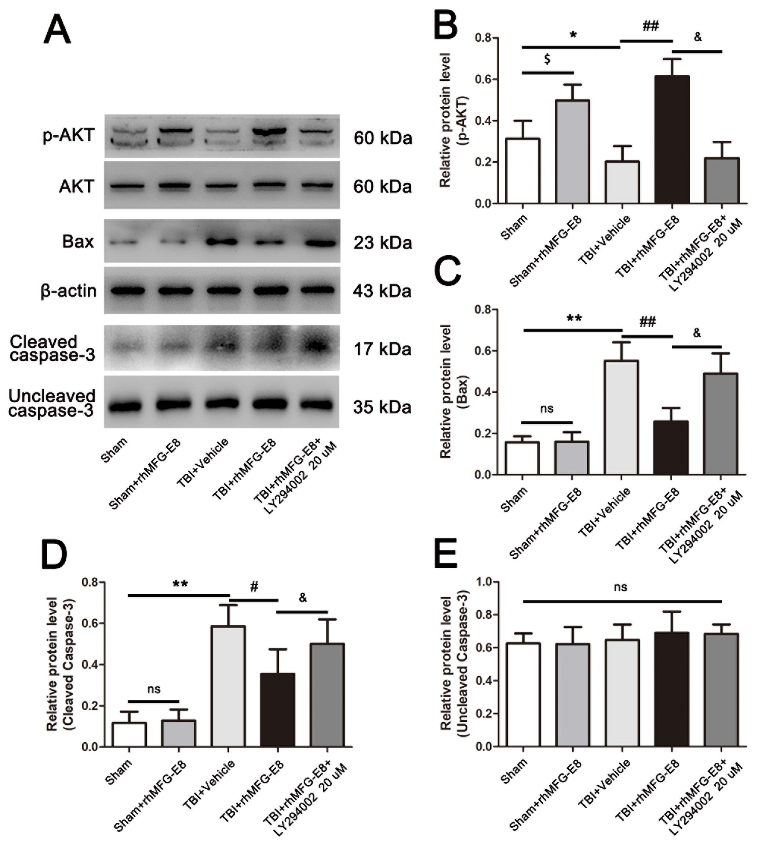


**Supplementary Figure 7**. Inhibited the PI3K/AKT signaling pathway, the neuroprotective effect of rhMFG-E8 on anti-apoptosis was evaluated by Western Blotting at 24 h after TBI. The results showed that treatment with rhMFG-E8 had no effect on the protein levels of Bax (C), cleaved caspase-3 (D) and uncleaved caspase-3 (E) as compared with Sham group, while the expression of p-AKT (B) was increased. Administration of LY294002 (PI3K inhibitor, 20 μM) dramatically decreased the expression of p-AKT, meanwhile abolished the effect of rhMFG-E8 on anti-apoptosis after TBI. The quantitative data are the mean ± SD (n = 6, each; ^$^*P* ＜ 0.05 vs. Sham group; ^*^*P* ＜ 0.05, ^**^*P* ＜ 0.01 vs. Sham group; ^#^*P* ＜ 0.05, ^##^*P* ＜ 0.01 vs. TBI + Vehicle group; ^&^*P* ＜ 0.05 vs. TBI + rhMFG-E8 group; ^ns^*P* ＞ 0.05 ).
